# Metal-organic and covalent organic frameworks for precision drug delivery: a precision-by-design evidence framework for biologically accountable nanomedicine

**DOI:** 10.3389/fddev.2026.1970554

**Published:** 2026-10-13

**Authors:** Xinwei Li, Haonan Zhang, Ruidong Ding, Feng Wang

**Affiliations:** 1 Organ Transplantation Center, The Affiliated Hospital of Qingdao University, Qingdao, Shandong, China; 2 University of Chinese Academy of Sciences, Beijing, China

**Keywords:** biomimetic nanomedicine, covalent organic frameworks, metal-organic frameworks, nanozyme, precision drug delivery, precision-by-design, stimuli-responsive release, theranostics

## Abstract

**Introduction:**

Metal-organic frameworks and covalent organic frameworks offer a rare opportunity to design drug carriers from molecular building blocks upward. Yet high loading capacity and stimulus-responsive release alone do not constitute precision delivery.

**Methods:**

We critically synthesized evidence on MOF and COF pore-level cargo interactions, framework and surface engineering, endogenous and externally triggered release, active targeting, biomimetic coatings, nanozyme activity and theranostic integration. Precision delivery was decomposed into five evidence-qualified dimensions: material, spatial, temporal, mechanistic and translational precision.

**Results:**

The evidence supports the complementary strengths of the two families across small-molecule, protein, nucleic-acid, gas and catalytic therapies. Direct evidence is strongest for individual component technologies, selected antibody-targeted systems and two-component hybrids. Recurrent evidence gaps include incomplete particle characterization in biological media, reliance on total-tissue accumulation rather than cell-specific delivery, and insufficient separation of carrier degradation from drug release.

**Discussion:**

A framework becomes precise only when it maintains a defined material identity, reaches a biologically justified compartment, releases an active payload at the intended place and time, and does so with measurable improvements in exposure, efficacy and safety. This requires a precision-by-design framework that maps claims to minimum evidence and links material critical quality attributes to biological mechanisms, translational assays and development decisions.

## 1 Introduction

Drug delivery is often described as a problem of transporting a therapeutic molecule from a dosage form to a diseased tissue. Precision drug delivery is more demanding. It requires control over a sequence of coupled events: formulation stability before administration, survival in the relevant biological fluid, distribution across organs, engagement with the intended cell population, intracellular trafficking where necessary, release of pharmacologically active cargo, and termination or clearance of both cargo and carrier. Failure at any link can erase an advantage established at another. A particle may load a drug efficiently yet release it prematurely in plasma; it may accumulate in a tumor while remaining trapped in stromal phagocytes; or it may enter target cells without releasing functional macromolecular cargo. These distinctions have become central to modern nanoparticle engineering and to critical analyzes showing that only a median of roughly 0.7% of the injected dose reaches solid tumors and that delivery efficiency is strongly conditioned by biological barriers rather than by material performance in buffered solution alone (Vargason et al., 2021; Mitchell et al., 2021; Wilhelm et al., 2016; Blanco et al., 2015).

MOFs and COFs are attractive in this context because they are reticular materials: extended networks are assembled from molecularly defined components, producing ordered pores and a high density of addressable chemical sites. The first porous crystalline COFs established that organic building blocks could be linked into ordered, permanently porous structures (Côté et al., 2005), while the development of MOFs from prototype zinc-carboxylate networks such as MOF-5 onward demonstrated how metal nodes and organic linkers can generate an immense range of topologies, functions and host-guest environments (Furukawa et al., 2013). Nanoscale porous MOFs were subsequently developed as carriers for drugs and imaging agents, providing early evidence that framework composition, pore volume and degradation could be used together in biomedical design (Horcajada et al., 2010), (Horcajada et al., 2011). These principles distinguish frameworks from non-porous inorganic particles and from many kinetically assembled soft carriers. The carrier is not merely a container: its node, linker, bond, defect, pore wall and external surface may each participate in loading, release, catalysis, imaging or biological recognition.

The same modularity also creates a risk of conceptual overreach. A large Brunauer–Emmett–Teller surface area measured on an activated powder does not directly predict usable loading in a hydrated nanosuspension. Acid-triggered disassembly in a simple buffer does not prove selective intracellular release. Fluorescence in a target organ does not establish intact-particle delivery, target-cell uptake or functional drug exposure. In addition, the word “biocompatible” is frequently assigned after short-term viability or histology experiments even though framework degradation can generate metal ions, ligands, fragments and re-coordinated species with distinct kinetics and toxicological profiles (Baati et al., 2013; Jing et al., 2026; Abbas et al., 2026; Sezgin et al., 2025). A precision claim must therefore be evaluated at the level of the complete product and its transformation over time, not inferred from the reputation of an individual building block.

Recent reviews have catalogued MOF synthesis, loading strategies, responsive systems and biomedical applications (He et al., 2021; Liu et al., 2024; Hu et al., 2025; Zhu et al., 2026), whereas parallel syntheses have mapped the rapidly expanding use of COFs in drug delivery, phototherapy, gene delivery and theranostics (Irie et al., 2026; Shi et al., 2023; Rathi et al., 2025; Zhong et al., 2025; Hu W. et al., 2026; Liu W. et al., 2022). The field is now mature enough to move beyond class-by-class inventories. The important question is not whether MOFs or COFs can carry drugs; many studies show that they can. The useful question is which framework attributes can solve a defined delivery bottleneck, how those attributes should be measured, and what evidence is required before the term precision is justified. In contrast to those catalogues, this Review contributes not another inventory of systems but an evidence framework: it converts “precision” from an aspirational label into five testable dimensions and a claim-to-evidence mapping (Table 1) applicable across material families and disease contexts. This shift also clarifies the complementary nature of the two material families. MOFs offer chemically diverse metal centers, coordination-bond lability, catalytic and imaging functions, and substantial experience with nanoscale synthesis. COFs offer metal-free or metal-sparse backbones, conjugated and photoactive skeletons, covalently encoded pore chemistry, and emerging control of two- and three-dimensional architectures. Neither family is intrinsically more precise. Precision emerges from matching the framework to the cargo, route, biological barrier and intended pharmacology.

**TABLE 1:** Claim-to-evidence criteria for precision delivery.

Claim	Minimum evidence	Common overinterpretation	Essential control
Targeted delivery	Quantitative target-cell exposure at stated resolution	Organ fluorescence equals target-cell delivery	Target-negative cells, receptor blocking, exposure-matched control
Functional macromolecule delivery	Sequence-, activity- or rescue-based endpoint	Fluorescent uptake equals functional release	Mismatch/rescue, endosomal escape, intact-cargo assay
Biodegradable carrier	Intact framework, fragments and excretion tracked over time	Loss of PXRD or fluorescence equals clearance	Dual labels or orthogonal component analysis
Synergistic therapy	Dose- or exposure-matched interaction analysis	Combination outperforms either single component	Physical mixture and inactive-structure controls
Theranostic system	Imaging/sensing informs localization, activation, response or dosing	Particle is fluorescent so it is theranostic	Signal calibration against chemical quantification

This Review develops a multiscale framework for that matching exercise. We first define precision delivery as a product of five linked dimensions: material identity, spatial targeting, temporal control, mechanistic matching and translational accountability. We then compare MOF and COF design spaces; analyze cargo loading, protection and release; discuss targeting, biomimetic interfaces and biological barriers; and examine endogenous and externally activated systems. We integrate catalytic nanozymes and hybrid extracellular-vesicle systems because they illustrate a transition from passive carriage to coordinated drug-and-material pharmacology. Finally, we propose critical quality attributes, evidence tiers and decision rules that can support reproducible development. The central position is that reticular programmability is valuable only when it is converted into a measurable improvement along the delivery chain, with explicit boundaries around what remains untested.

### 1.1 Scope and evidence approach

This is a narrative, mechanism-organized Review rather than a systematic review or meta-analysis. Literature was discovered through searches centered on MOF and COF drug delivery, responsive release, biological barriers, biomimetic coatings, nanozyme therapy, pharmacokinetics, degradation, toxicology and manufacturing, with coverage through 14 August 2026. Foundational reticular-chemistry papers were retained to explain material principles; recent reviews were used for field-level synthesis; and primary studies were prioritized for statements about loading, targeting, release mechanisms or therapeutic effects. The evidence base is dominated by cell and small-animal research. Accordingly, improvements in tumor growth, metabolic readouts, oxidative injury or tissue repair are described as preclinical findings rather than as evidence of clinical benefit.

To make this evidence-gathering process reproducible, literature was identified through PubMed/MEDLINE, Web of Science, Scopus and Crossref using combinations of the terms ‘metal-organic framework’, ‘covalent organic framework’, ‘drug delivery’, ‘responsive release’, ‘biological barrier’, ‘biomimetic’, ‘nanozyme’, ‘pharmacokinetics’, ‘degradation’, ‘toxicology’ and ‘manufacturing’, with coverage through 14 August 2026 and no language restriction. Records were screened by title and abstract against the review’s scope; approximately 1,200 records were identified and approximately 150 primary studies and reviews were retained, prioritizing primary data for mechanistic or efficacy claims and recent reviews for field-level synthesis. Because this is a narrative review rather than a systematic review or meta-analysis, no formal risk-of-bias tool was applied; the non-systematic nature of this review is stated explicitly in the Limitations of this Review section.

Claims are graded implicitly by the endpoint measured. Material feasibility means that a framework can be synthesized, loaded or triggered under specified conditions. Delivery feasibility requires protection, transport and release in a biologically relevant model. Pharmacological delivery requires a target-engagement or functional-cargo readout, not fluorescence alone. Translational evidence additionally requires interpretable pharmacokinetics, biodistribution, degradation, toxicology, product definition and manufacturing control. Reviews of commercial delivery technologies and precision nanoparticles provide a useful comparator: successful delivery platforms tend to reduce formulation complexity to attributes that can be controlled and linked to clinical function (Vargason et al., 2021; Mitchell et al., 2021). The inclusion of extracellular vesicles and cell-derived interfaces is limited to cases where they directly inform framework hybridization, barrier navigation or cargo function (Herrmann et al., 2021; Wang L. L.-W. et al., 2024).

Six publications from the authors’ research program are discussed where they support the topic directly or provide a clearly labelled adjacent design lesson. These include MOF-exosome composites for multiorgan fibrosis, MOF-818 and ASGR1-directed platforms for steatotic liver disease, an integrated nanozyme-exosome system, GPX4-containing exosomes in steatotic liver ischaemia-reperfusion injury, and a bimetallic MOF nanozyme for sensing-assisted diabetic wound treatment (Liu et al., 2026; Zhang et al., 2026; Li et al., 2025; Yu et al., 2026; Shi et al., 2025; Yu et al., 2025). Three other author publications were excluded because their principal questions concern food-quality sensing, circRNA liquid biopsy or microbiota-mediated transplant immunology rather than MOF/COF precision delivery. This selective approach avoids using self-citation as a substitute for topic relevance.

## 2 What makes framework delivery precise?

Precision delivery should be treated as a claim about a complete drug product rather than as a synonym for active targeting. We define five linked dimensions: material precision, spatial precision, temporal precision, mechanistic precision and translational precision. Each dimension has a different evidentiary burden, and failure at one stage can erase an apparent advantage at another.

Material precision concerns reproducible composition, crystallinity, defect state, cargo state, hydrated size, aggregation and surface identity. Spatial precision concerns exposure at the stated organ, tissue, cell or subcellular level. Temporal precision concerns suppression of leakage during transit and activation over a pharmacologically meaningful window. Mechanistic precision concerns alignment between framework-derived function, cargo action and a validated disease vulnerability. Translational precision concerns whether these relationships can be measured, reproduced and controlled during development. These five dimensions map onto the quality-by-design vocabulary of pharmaceutical development: material precision corresponds to critical quality attributes (CQAs), the process controls that deliver them to critical process parameters (CPPs), mechanistic precision to the link between a CQA and the mode of action, and translational precision to the ability to hold a quality target product profile (QTPP) constant from candidate selection through pivotal studies.

The five dimensions should not be scored by isolated material metrics. Reducing particle size may improve dispersion while accelerating dissolution or changing clearance; a coating may improve serum stability while masking pores or ligands; and a targeting ligand may increase receptor-mediated uptake while shortening circulation. The appropriate design objective is therefore a constrained operating window in which the final formulation completes the intended delivery sequence.

Precision claims must be stated at the resolution actually measured. Whole-organ fluorescence supports organ-associated signal, not intact-particle delivery to a target cell. Cell uptake does not establish cytosolic release, and intracellular localization does not establish functional protein, RNA or enzyme activity. A useful evidence sequence is: intact-product stability, quantitative exposure, cell-resolved uptake, release or framework transformation, functional cargo or catalytic activity, pharmacodynamic response and component-resolved safety. This multiscale evidence chain is summarized in Figure 1.

**FIGURE 1:**
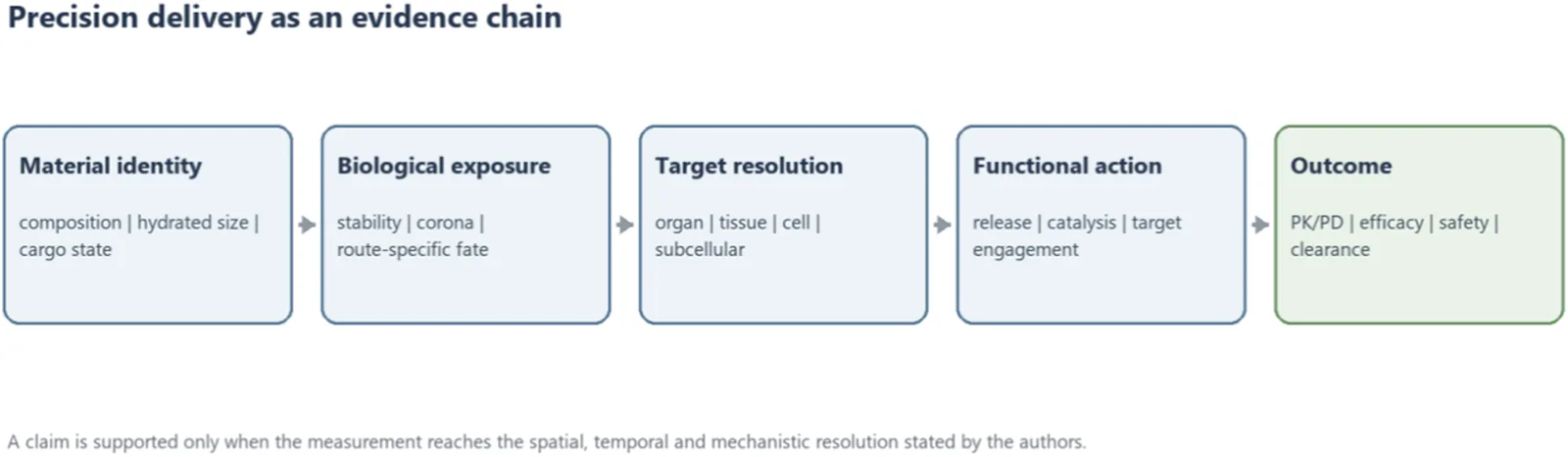
A multiscale definition of precision framework delivery. A left-to-right evidence chain connects (i) molecular and particle identity, (ii) stability in the dosage form and biological fluids, (iii) route-specific biodistribution, (iv) cell and subcellular engagement, (v) triggered release or framework-derived activity, and (vi) pharmacological effect, safety and clearance. Parallel tracks show the required measurements for MOFs (metal/linker/cargo) and COFs (backbone fragments/cargo/coating). Red warning symbols mark common inferential gaps: dry porosity substituted for hydrated loading, organ fluorescence substituted for target-cell uptake, uptake substituted for functional cargo release, and short-term histology substituted for product safety. The molecular end of the chain should be anchored by representative MOF node/linker and COF linkage structures from Section 3. This original schematic is provided for the present Review and does not reuse published artwork.

The same logic applies to temporal and mechanistic claims. A release curve in a simple buffer is not evidence of site-selective release, and a combination that outperforms either component is not automatically synergistic. Studies should use protein-containing and non-sink conditions where relevant, physiologically attainable triggers, framework-only and cargo-only controls, dose-equivalent comparisons and an interaction model when synergy is claimed.

We therefore use the term precision as an evidence-qualified descriptor. A targeted carrier requires quantitative receptor- or ligand-dependent delivery at the claimed spatial scale; a biodegradable carrier requires identification and time-resolved disappearance of the intact framework plus fate of its fragments; a theranostic system requires that the diagnostic output informs localization, activation, response or dosing. This claim-to-evidence mapping is the basis for the decision framework developed in Sections 10–12.

## 3 Reticular material space: complementary strengths of MOFs and COFs

### 3.1 MOFs: coordination chemistry as carrier and active component

MOFs are assembled from metal ions or clusters (secondary building units, SBUs) connected by multidentate organic linkers. Node identity, coordination geometry, linker functionality, defect state and particle architecture jointly determine hydrated stability, pore accessibility, degradation and framework-derived activity. These variables should be treated as product attributes rather than inferred from a framework name. Among the families most relevant to drug delivery, zirconium frameworks such as UiO-66 are built from highly connected Zr_6_O_4_(OH)_4_ clusters whose strong Zr(IV)–carboxylate bonds confer exceptional hydrolytic and thermal stability; ZIF-8 assembles zinc and 2-methylimidazolate into a sodalite-type network that is comparatively stable near neutral pH but dissolves once the imidazolate linkers are protonated at low pH; MIL-family iron carboxylates are assembled from Fe_3_O clusters and are designed for degradability; and cyclodextrin MOFs coordinate potassium with γ-cyclodextrin to form edible, bioabsorbable carriers. Porphyrinic Zr-MOFs such as PCN-224 and PCN-222 additionally embed a photosensitizing porphyrin directly into the framework, merging carrier and photodynamic function. Each framework name nonetheless covers formulations with potentially different size, surface chemistry, modulator residues and biological fate (Horcajada et al., 2010; Horcajada et al., 2011; He et al., 2021; Liu et al., 2024; Hu et al., 2025; Zhu et al., 2026).

Coordination lability is simultaneously an enabling feature and a liability. Dilution, phosphate, proteins and competing ligands can destabilize a framework before it reaches the intended compartment, whereas a highly stable framework may retain cargo or persist in organs. Node selection must therefore be linked to speciation: intact particles, soluble ions, protein-bound metals and linker-derived fragments may have different exposure and toxicity. Nutritional familiarity with a free element cannot establish the safety of the corresponding framework.

MOF cargo integration includes pore adsorption, one-pot encapsulation, coordination to nodes, covalent attachment, surface adsorption and growth around a cargo. Biomimetic mineralization is particularly useful when a protein or nucleic acid exceeds the pore aperture, but it should be described as protective encapsulation rather than conventional pore loading (Adhikari and Chakraborty, 2016; Liang et al., 2015). The relevant design question is whether the cargo must remain accessible, be released extracellularly or reach the cytosol after framework dissolution.

MOFs may also be pharmacologically active through redox chemistry, gas generation, imaging, enzyme confinement or nanozyme catalysis (Fang et al., 2020; Guan et al., 2023; Wu et al., 2023; Duan et al., 2023; Jiang et al., 2023; Xiang et al., 2023). This expands the therapeutic design space but creates a multi-entity dose problem. Particle mass, metal content, cargo amount and catalytic activity may all be relevant, and the active entity may change as the framework transforms. Framework-derived activity should therefore be measured independently from cargo activity and normalized to the dose metric used *in vivo*.

### 3.2 COFs: covalent pore chemistry and photophysical function

COFs are constructed from organic building blocks joined by covalent linkages into crystalline two- or three-dimensional networks. Linkage chemistry determines the balance between dynamic bond formation during crystallization and stability during formulation or use. Boronate and boroxine linkages, imine, hydrazone, azine, imide, beta-ketoenamine and related chemistries should not be grouped under a single stability label (Côté et al., 2005; Liu et al., 2021; Kandambeth et al., 2018). Hydrolytic stability generally increases along the series boroxine and boronate ester < imine < hydrazone and azine < β-ketoenamine < imide < sp^2^-carbon and olefin-linked backbones, with β-ketoenamine gaining robustness from enol–keto tautomerization and intramolecular hydrogen bonding. This ordering, not a generic “COF stability” label, should guide the choice of linkage for a given route and release mechanism.

COFs offer aromatic pore walls, tunable electronic structures and, in conjugated systems, intrinsic light-harvesting or photoactive functions. These attributes can support hydrophobic drug binding, photodynamic or photothermal therapy and short diffusion paths in two-dimensional particles. They can also promote stacking, hydrophobic aggregation or pore inaccessibility in physiological media. Three-dimensional COFs provide interconnected channels and large pore volumes but remain more challenging to prepare as uniform, dispersible nanoparticles (Fang et al., 2015; Bai et al., 2016; Zhang et al., 2018; Jia et al., 2020; Tong et al., 2020; Ge et al., 2021; Anbazhagan et al., 2021; Li M. et al., 2021; Zhou et al., 2022; Zhao et al., 2023; Das et al., 2022).

Beyond their photophysical function, COFs can also act as intrinsic nanozymes, with their periodic structure and tailorable functionalities providing enzyme-mimetic catalytic activity (Hu B. et al., 2026).

Metal-free composition reduces some metal-ion liabilities but does not establish biological safety. Monomers, catalysts, solvents, hydrophobic surfaces, persistent backbones and degradation fragments all require product-level evaluation. A deliberately degradable COF may improve clearance but compromise plasma stability or storage, whereas a robust COF may remain intact beyond the therapeutic window (Zhong et al., 2025; Hu W. et al., 2026), (Zhao et al., 2026; Kolodziejczyk and Plonska-Brzezinska, 2026; Li et al., 2023; Kaur et al., 2024). The central comparison with MOFs is therefore not metal versus no metal, but which material offers a better controlled balance of function, fate and analytical tractability.

### 3.3 Choosing between, or combining, framework families

Selection between MOF and COF should begin with the delivery bottleneck. MOFs may be favored when coordination chemistry, metal-center catalysis, metal-ion output, node-mediated degradation or metal-associated imaging is necessary. COFs may be favored when a metal-free or metal-sparse pore environment, covalently encoded trigger chemistry, conjugated photophysics or tunable aromatic interactions is the decisive function. Neither choice is inherently more precise; precision is established only by the final formulation and its biological evidence. A head-to-head comparison of the two framework families is provided in Table 2.

**TABLE 2:** Head-to-head comparison of MOFs and COFs for precision delivery.

Attribute	MOFs	COFs	Implication for precision design
Backbone/building blocks	Metal nodes (SBUs) + organic linkers	Covalently linked organic monomers	Node lability vs. covalent-bond choice sets the degradation-tuning knob
Hydrolytic stability	Tunable over a wide range (labile ZIF-8 to robust UiO-66)	Generally high; tunable by linkage choice	Match stability to route and intended release mechanism
Degradation products	Metal ions, linkers and re-coordinated species	Organic monomers/oligomers and backbone fragments	Different toxicology and clearance questions require different assays
Analytical traceability	Metal traceable by ICP-MS for quantitative PK	Metal-free backbone is harder to trace; needs isotopic or dual-label strategies	COF fate is analytically more demanding than MOF fate
Cargo scope	Small molecules, biomacromolecules (biomineralization), gases	Predominantly small molecules; aromatic pore-wall binding	Match cargo to pore/wall chemistry rather than forcing one family
Intrinsic function	Metal catalysis, redox, magnetism, imaging	Conjugated photophysics, PDT/PTT, fluorescence	Choose the family by the framework-derived activity actually required
Scalability/process	More mature nanosynthesis; modulator control established	2D facile; uniform 3D nanoparticles harder	Manufacturing burden differs by family and must be planned early
Primary regulatory risk	Metal accumulation and ion toxicity	Persistent backbone, monomer/aldehyde residues	Different impurity and safety strategies per family

A decision should compare hydrated porosity, cargo affinity, serum stability, corona formation, degradation products, target-cell exposure, dose definition, manufacturing burden and benchmark performance. MOF-on-MOF, MOF@COF, COF@MOF and membrane or vesicle hybrids are justified only when the interface creates a measurable gain that a single framework or simpler carrier cannot provide. Added components should be linked to critical quality attributes and modular potency assays. Table 3 summarizes the precision-by-design variables, their minimum characterization and decision-oriented assays, and Figure 2 provides a selection matrix relating cargo class, biological barrier and activation mechanism.

**TABLE 3:** Precision-by-design variables for MOF and COF delivery systems.

Design variable	Precision function	Minimum characterization	Common failure mode	Decision-oriented assay
Node or linkage chemistry	Controls stability, degradation and catalytic activity	Composition, oxidation state, linkage conversion, residual reagents	Assuming nominal composition equals biological speciation	Time-resolved degradation and component speciation in route-relevant media
Pore size and pore-wall chemistry	Controls cargo affinity, loading state and diffusion	Gas sorption plus hydrated-state or cargo-loaded structural analysis	Using dry BET area as a surrogate for usable biological porosity	Loading isotherm, binding analysis and release under sink/non-sink conditions
Particle size, shape and aggregation	Influences circulation, uptake, penetration and clearance	Number- and mass-weighted size, morphology, polydispersity in formulation and biofluids	Reporting only dry TEM size	Time-resolved DLS/NTA, microscopy and fractionation in serum-containing media
Defects and modulators	Tune porosity and reactivity	Defect markers, modulator and solvent residues, batch distribution	Uncontrolled defects change dissolution and toxicity	Batch-correlated degradation, potency and impurity testing
Cargo location and physical state	Determines protection and release mechanism	Encapsulation efficiency, loading content, spatial localization, crystallinity and activity	Surface-adsorbed cargo mistaken for pore encapsulation	Orthogonal extraction, spectroscopy, microscopy and activity assays
External surface chemistry	Controls colloidal stability, corona and ligand accessibility	Ligand density, orientation, charge, hydrophobicity and coating integrity	Targeting ligand is buried or shed *in vivo*	Quantitative ligand-binding and receptor-blocking studies after biofluid exposure
Stimulus response	Provides temporal and local release	Trigger threshold, kinetics, reversibility and off-target activation	Trigger conditions are not physiologically attainable	Release and potency under matched pathological and normal conditions
Framework-derived activity	Adds catalytic, photophysical or immunological action	Activity per mass and per metal/linker dose, substrate dependence	Carrier activity confounded with cargo effect	Framework-only, cargo-only, mixture and inactive-framework controls
Degradation products	Determines clearance and delayed risk	Identity, kinetics, protein binding and excretion	Intact-particle signal cannot be separated from fragments	Dual-labelled or orthogonal metal/linker/cargo mass balance
Manufacturing attributes	Enables reproducibility and scale-up	Yield, size distribution, loading, sterility, endotoxin and storage stability	Laboratory batch cannot be translated to controlled process	Defined release specifications and stability-indicating assays

**FIGURE 2:**
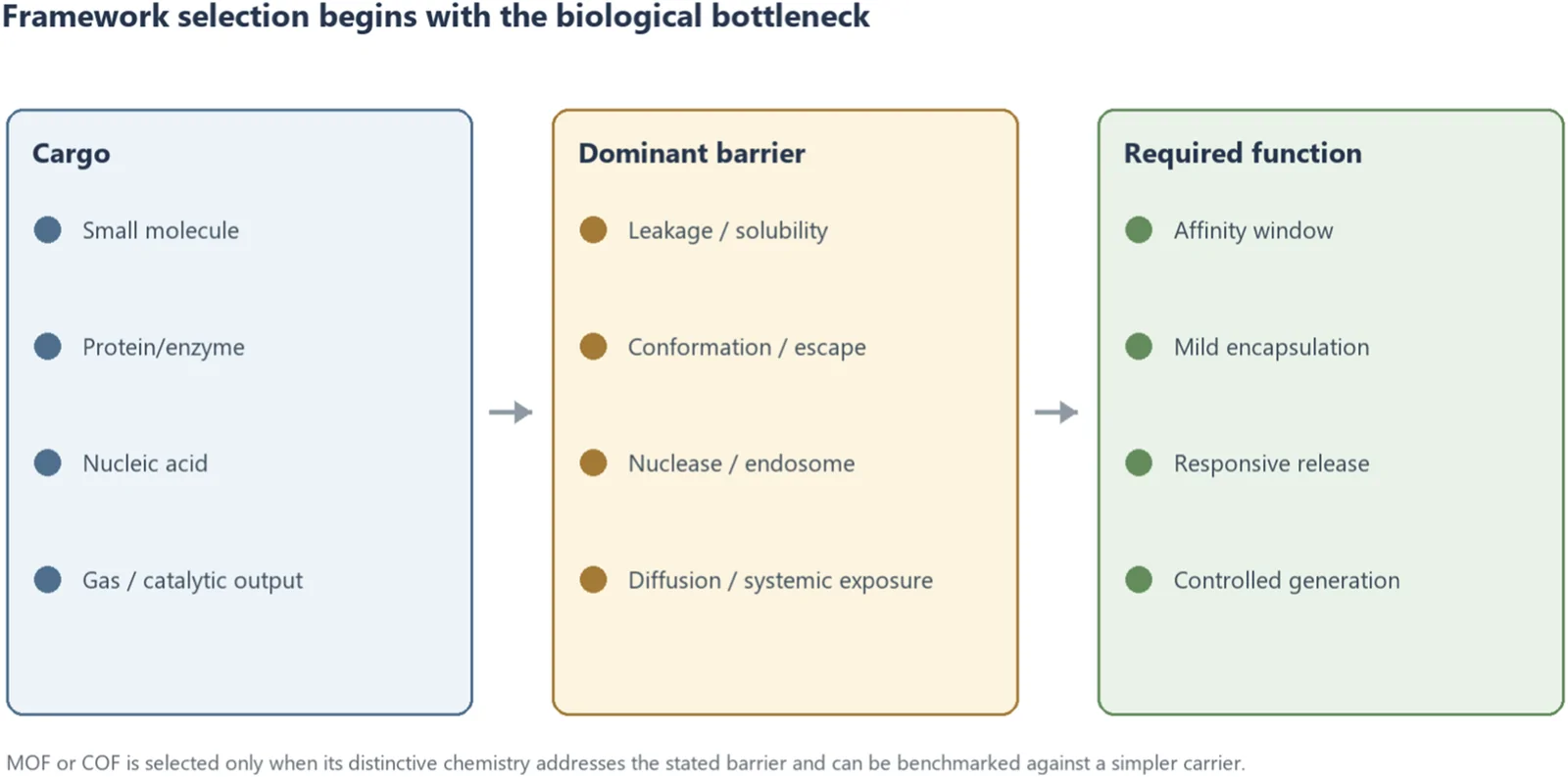
Framework selection matrix for cargo, barrier and activation mechanism. A three-axis matrix maps cargo class (small molecule, protein/enzyme, nucleic acid, gas or catalytic substrate), dominant delivery barrier (systemic clearance, extracellular matrix, endosome, mucosa or local tissue retention), and desired activation mechanism (framework dissolution, cleavable gate, enzyme/metabolite, light, ultrasound/magnetic field or constitutive catalysis). MOF and COF icons occupy cells only when their chemical strengths address the barrier. Side panels show recommended comparators and functional endpoints. The schematic emphasizes that platform selection begins from the biological bottleneck rather than from a favorite framework.

## 4 Cargo integration: loading is not delivery

### 4.1 Small molecules and the problem of reversible affinity

Small molecules remain the largest application class because they can enter pores and interact with linkers or nodes through hydrophobic forces, hydrogen bonding, electrostatics, pi-stacking or coordination. High loading is not the design endpoint. The relevant objective is an affinity window that limits leakage during transit while permitting pharmacologically useful release at the intended site.

One-pot assembly can reduce post-loading loss, whereas post-synthetic adsorption simplifies formulation and permits loading of a pre-characterized framework (Adhikari and Chakraborty, 2016). Surface-bound and pore-bound cargo populations may have different release kinetics. Coordination or covalent attachment can improve retention but may create exchange reactions or a new chemical entity. Each loading mode should therefore be verified by orthogonal localization, free-fraction, release and activity measurements.

The physical state of the cargo is also part of the product definition. Confinement may maintain an amorphous or molecularly dispersed drug, but metastable states can recrystallize during storage or release. Cyclodextrin-MOF systems illustrate the possible combination of host–guest inclusion, porosity and surface engineering (Jia et al., 2019; Nie et al., 2023). Their value should be tested against simpler cyclodextrin complexes, amorphous dispersions, liposomes or polymer systems rather than against an unoptimized free-drug control alone.

COF systems make the affinity–release trade-off especially visible because aromatic pore walls can strongly bind anthracyclines and other planar drugs (Fang et al., 2015; Bai et al., 2016). Redox-, acid- and dual-responsive systems combine retention with triggered disassembly (Anbazhagan et al., 2021; Fu et al., 2024; Liu et al., 2020). These studies should distinguish release caused by bond cleavage from release caused by swelling, aggregation, pore blockage or nonspecific erosion. A responsive label is meaningful only when the trigger is attainable in the intended biological compartment.

### 4.2 Proteins, enzymes and nucleic acids

Proteins, enzymes and nucleic acids create a separate design problem because their dimensions, conformation and functional compartment matter. Biomimetic mineralization can protect macromolecules under mild aqueous conditions (Liang et al., 2015), but high encapsulation does not establish retained activity. The formulation must be evaluated for conformational integrity, protection during transit, release after uptake and endosomal escape where cytosolic action is required.

Enzyme-loaded MOFs can act as confined bioreactors, but catalytic cascades depend on mass transport and the availability of oxygen, glucose, proton donors or other substrates *in vivo* (Fang et al., 2020). Initial activity should be complemented by catalytic half-life, activity across physiological substrate ranges, serum interference and framework-only, enzyme-only and inactive-framework controls. Co-localization should not be called synergy without dose-matched interaction analysis.

Nucleic-acid delivery requires protection from nucleases, uptake, endosomal escape and functional release. Fluorescent uptake or reporter expression alone is insufficient. A rigorous package should quantify intact cargo, endosomal escape, cytosolic availability, sequence-specific target modulation and a rescue or mismatch control (Mitchell et al., 2021; Blanco et al., 2015). The same claim-to-evidence logic applies to protein, enzyme and genome-editing cargos.

For vaccines, precision includes antigen integrity, adjuvant activity, lymphatic trafficking, antigen-presenting-cell exposure and the quality and duration of innate and adaptive responses. Cyclodextrin MOFs have been evaluated as vaccine adjuvants (Li C. et al., 2021), but repeat dosing, metal-dependent inflammation and long-term particle fate remain important boundaries. These systems should be discussed as immune-active formulations rather than as passive carriers.

### 4.3 Gases, ions and catalytic equivalents as non-conventional cargos

Therapeutic gases such as nitric oxide, carbon monoxide and hydrogen sulfide require local concentration–time control because their effects are highly dose- and context-dependent (Zhou et al., 2021). Gas adsorption measured under high pressure is not equivalent to a useful dose in hydrated tissue. Studies should quantify loading, release, diffusion away from the target and systemic haemodynamic or respiratory consequences.

Metal ions and catalytic products are better classified as pharmacological outputs when they are generated during framework transformation. Iron, copper, cerium and multimetal centers can shift between pro-oxidant and antioxidant behavior depending on valence, pH, substrate and biomolecular context. Intact particles, soluble metals, protein-bound fractions and downstream redox products should be tracked together so that degradation is not mistaken for clearance or catalytic action.

### 4.4 Route-specific cargo design: oral, pulmonary and depot delivery

Route-specific design should be evaluated using route-specific barriers and comparators. Oral delivery must account for fasted and fed gastric states, bile salts, enzymes, mucus, epithelial transport and microbiota rather than a single simulated gastric-fluid experiment. The key endpoint is active drug exposure at the intended intestinal compartment, not merely particle survival in buffer.

For insulin and other proteins, protection of structure and bioactivity is more informative than immunoreactive recovery alone. An injectable MOF-based *in situ* gel illustrates local protection and responsive release (Liu Y. et al., 2022), whereas oral delivery would additionally require epithelial access, appropriate release location and route-specific toxicity. Glucose and pH should be modelled over repeated dosing when they are part of the trigger mechanism.

Pulmonary delivery imposes constraints on aerodynamic diameter, emitted dose, redispersibility, regional deposition and macrophage interaction. Gamma-cyclodextrin MOFs provide an example of route-specific formulation (Nie et al., 2023), but the powder must be characterized after aerosolization. Chronic use requires assessment of mucociliary clearance, alveolar macrophage burden, local inflammation and the fate of soluble metal, linker and nanoscale fragments.

Depot delivery can reduce broad biodistribution but does not eliminate systemic exposure. Hydrogels, implants, dressings and microneedles couple swelling, erosion, diffusion and framework degradation, so release should be tested using the complete formulation rather than isolated particles (Sivakumar et al., 2017). Local retention, tissue exposure, soluble components and wound or tissue recovery should be measured together.

The benchmark must follow the route: an oral framework should be compared with established solubilization or enteric-protection technology, an inhaled framework with a suitable dry-powder or nebulized product, and a depot with polymer-only controlled release. Framework complexity is justified only when it provides a route-relevant gain in active exposure, protection, dosing frequency, local activation or therapeutic index. Cargo-specific evidence packages are summarized in Table 4.

**TABLE 4:** Cargo-specific evidence packages.

Cargo/output	Dominant barrier	Functional endpoint	Minimum controls
Small molecule	Affinity–leakage–release balance	Free-drug exposure and pharmacodynamic effect	Free drug, simple formulation, free fraction
Protein/enzyme	Conformation, protection and compartment access	Recovered activity or target engagement	Structure, activity, escape and framework-only
Nucleic acid	Nuclease stability and endosomal escape	Sequence-specific modulation or rescue	Mismatch, intact cargo, cytosolic delivery
Therapeutic gas	Concentration–time profile and diffusion	Local pharmacology with systemic safety	Hydrated release, haemodynamic/respiratory endpoints
Catalytic output	Substrate availability and off-target reaction	Dose-normalized catalytic activity and PD effect	Free metal, inactive material, serum interference

## 5 Spatial precision: biological barriers, targeting and biomimetic interfaces

### 5.1 From administration site to target cell

The barrier sequence depends on route. Intravenous particles encounter dilution, salts, proteins, complement, blood cells, vascular endothelium and the mononuclear phagocyte system. Oral systems face acid, digestive enzymes, mucus, food effects and epithelial transport. Pulmonary systems must navigate aerosolization, mucus, surfactant and macrophage clearance. Local hydrogels, wound dressings and microneedles reduce systemic barriers but introduce diffusion, tissue retention and device-material interactions. Framework design should therefore start with a route-specific failure map rather than a generic statement that nanoscale size improves bioavailability.

For systemic delivery, size and surface chemistry jointly influence circulation and organ capture. Protein adsorption can mask a targeting ligand, change hydrodynamic size and create new receptor interactions. PEGylation of UiO-66 improved colloidal stability and altered cellular uptake while preserving pH-responsive release (Abánades Lázaro et al., 2017), illustrating how an external polymer layer can tune several delivery steps. Yet PEG density and conformation must be quantified, and repeated exposure may produce anti-PEG responses in some contexts. Alternative hydrophilic polymers, zwitterions, polysaccharides and biological membranes can reduce nonspecific interactions, but none is universally stealthy.

Tumor delivery remains a widely used test case and a frequent source of overstatement. Meta-analysis of nanoparticle delivery has shown that only a small fraction of administered dose typically reaches solid tumors (Wilhelm et al., 2016). The enhanced permeability and retention effect is heterogeneous across tumor type, size, vascular state and model. Active ligands usually improve cellular binding and internalization more reliably than they increase the fraction of injected dose reaching a tumor. Consequently, a convincing targeting study should report blood exposure, whole-organ distribution, tumor concentration, cell-type distribution within the tumor and competition or receptor-loss controls. It should also compare equal particle doses and ligand densities, because changes in formulation can alter pharmacokinetics independently of receptor recognition.

### 5.2 Molecular targeting and trigger-target coupling

Framework surfaces can display antibodies, peptides, aptamers, carbohydrates or small molecules. Aptamer-functionalized MOF nanoparticles have been designed for vascular endothelial growth factor-triggered drug release, coupling biomarker recognition to payload liberation rather than using recognition only for binding (Chen et al., 2018). Folic acid and other receptor ligands have also been integrated into multifunctional MOF systems (Wang et al., 2015). Such trigger-target coupling is attractive because it can suppress release in transit and activate it near a molecular cue. The challenge is specificity: many biomarkers are heterogeneous or present at lower levels in healthy tissues, while aptamer structure may change after conjugation or biofluid exposure. Ligand affinity should therefore be measured on the final particle, not inferred from the free ligand.

Targeting precision can be strengthened by matching receptor biology to the intended cell. The authors’ ASGR1-directed platforms provide an example in hepatocytes. A MOF-818-based nanozyme was modified with an ASGR1 antibody and loaded with uridine diphosphate glucose to coordinate antioxidant catalysis, lipid efflux and suppression of lipogenesis in preclinical metabolic dysfunction-associated steatotic liver disease (MASLD) models (Zhang et al., 2026). A related anti-ASGR1 nanocomplex combined a MIL-family core with thyroid hormone for rapid reduction of donor-liver steatosis, including evaluation in transplantation-associated perfusion (Li et al., 2025). These studies support receptor-informed organ and cell targeting, but they do not remove the need to quantify receptor saturation, off-target uptake, changes in ASGR1 expression across disease severity and the fate of antibody-bearing degradation products.

### 5.3 Biomimetic coatings and extracellular-vesicle hybrids

Cell membranes and extracellular vesicles can provide complex surface repertoires that are difficult to reproduce with a single ligand. A membrane coating may reduce premature immune recognition, enable homotypic interactions, bind inflamed endothelium or present naturally oriented proteins. In a cancer model, a mitochondria-targeted cancer-cell-membrane-biomimetic MOF combined sonodynamic therapy with checkpoint blockade and enhanced preclinical antitumor immunity (Luo et al., 2022). Such systems demonstrate functional possibilities, but a membrane is not a uniform excipient. Source-cell identity, culture state, purification, protein orientation, residual nucleic acids and batch variation become part of the product definition.

Extracellular vesicles offer both a biological membrane and an endogenous cargo compartment. Their emerging role as drug-delivery systems is supported by extensive engineering research (Herrmann et al., 2021). Framework-evoked hybridization can address complementary weaknesses: a MOF may protect vesicles, add pH-responsive release or catalytic activity, whereas the vesicle may contribute biologically derived cargos and membrane interactions. The authors’ MOF-engineered exosome system encapsulated exosomes with Cu-Cys nanozyme activity in a ZIF-containing architecture, added polydopamine and antibody modules, and reported preclinical benefit across lung, liver and kidney fibrosis models (Liu et al., 2026). The work illustrates how a hybrid can coordinate vesicle protection, catalytic ROS control, organ targeting and metabolic regulation. It also illustrates why hybrid potency assays must be modular: exosome cargo activity, nanozyme activity, release behavior and targeting function should each be measurable.

An integrated copper-nanozyme/exosome/ASGR1 platform for MASLD further combined catalytic antioxidant activity, exosomal regulatory cargo and hepatocyte targeting (Yu et al., 2026). A separate study of spindle-shaped urine-derived stem-cell exosomes showed that GPX4-containing vesicles could inhibit ferroptosis and alleviate steatotic liver ischaemia-reperfusion injury in preclinical models (Shi et al., 2025). Although the latter did not use a MOF or COF, it provides a relevant cargo-design lesson: vesicles can deliver functional proteins that might be stabilized or spatially controlled by a framework hybrid. Such a combination remains a proposal until directly tested. Citing the component studies does not establish the efficacy or safety of an untested integrated product.

Biomimetic interfaces also change the analytical burden. Conventional particle size and zeta potential are insufficient to confirm complete coating. Cryogenic or high-resolution imaging, protein and lipid profiling, protease accessibility, orientation-sensitive binding, membrane continuity and stability under shear may be needed. The field should distinguish membrane-coated particles from membrane-particle mixtures and determine whether the coating remains attached during circulation. A biologically complex shell can improve function, but only if its identity is reproducible and its contribution survives the route of administration.

## 6 Temporal precision: endogenous and externally controlled release

### 6.1 pH, redox and competitive-coordination triggers

Acid response is the most common trigger in framework drug delivery. ZIF-8 and other acid-labile structures can remain comparatively stable near physiological pH (∼7.4) and begin to disassemble as pH falls below roughly 5.5–6.0 in acidic endosomal or tumor-associated environments. COFs can incorporate imine, hydrazone or other acid-sensitive linkages, while surface gates may open after protonation. The conceptual advantage is clear: reduce leakage during circulation, then accelerate release after uptake. The experimental risk is equally clear. Many studies compare pH 7.4 with pH 5.0 in salt-poor buffers, whereas extracellular tumor pH is typically only moderately acidic (about pH 6.5–6.8) and protein-rich, while endosomal and lysosomal compartments reach roughly pH 4.5–5.5 only after cellular uptake. Release experiments should therefore include route-relevant media and explicitly state whether the intended trigger is extracellular or intracellular.

Redox-responsive systems exploit high intracellular glutathione or disease-associated ROS. Thioether, disulfide and redox-active metal components can alter framework stability or release (Anbazhagan et al., 2021; Liu et al., 2020). Dual-responsive designs aim to reduce false activation by requiring more than one signal. A light-activated hypoxia-sensitive COF is an example of tandem response, integrating an externally controlled input with a pathophysiological state (Ge et al., 2021). Logic-gated release is attractive, but each additional gate can slow or incompletely release the cargo. The system should be benchmarked against single-gate controls and tested across realistic distributions of pH, glutathione, oxygen and enzyme activity rather than at one idealized concentration.

Competitive coordination is especially relevant to MOFs. Phosphate, bicarbonate, amino acids and proteins can attack nodes or displace linkers. This behavior can be used intentionally for biodegradation, but it can also undermine circulation stability. A buffer that preserves a framework may not predict plasma stability, and serum alone may not reproduce interstitial or intracellular chemistry. Orthogonal measurements of particle structure, soluble metal, linker release and cargo release are needed to determine whether a response represents selective gating, gradual erosion or catastrophic collapse.

### 6.2 Enzymes, metabolites and self-amplifying reactions

Enzyme-responsive coatings can exploit matrix metalloproteinases, hyaluronidase, esterases or intracellular proteases. Metabolites such as glucose or ATP can act as substrates or competitors. The appeal is biological specificity, but enzyme levels vary among patients and within lesions. A delivery system should be characterized over a concentration range and in the presence of structurally related enzymes. If the trigger is a substrate-consuming cascade, the treatment may also alter the trigger while operating, producing nonlinear release or catalytic behavior.

Self-amplifying systems deliberately use this nonlinearity. Glucose oxidase can lower pH and generate hydrogen peroxide, promoting MOF degradation or Fenton-like catalysis (Fang et al., 2020). Hypoxia-sensitive components may become active as oxygen is consumed, and catalytic ROS can damage membranes to increase further penetration. These positive feedback loops can produce strong local effects but also make dose-response less predictable. Appropriate controls should measure substrate depletion, oxygen, pH, intermediate ROS, final products and cell death mechanism over time. A single terminal ROS probe cannot establish the proposed cascade.

### 6.3 Light, ultrasound, magnetic fields and heat

Externally applied energy provides temporal control and can be spatially focused. Porphyrinic MOFs and conjugated COFs can generate singlet oxygen for photodynamic therapy, convert light to heat, or release a drug after photochemical cleavage. COF nanosheets have been developed as photosensitizers with substantial cargo-loading capability (Tong et al., 2020), and nanoscale covalent organic polymers have served as carrier-free, biodegradable nanomedicines integrating chemotherapy with photodynamic therapy (Wang et al., 2018). CuS@COF nanosheets illustrate dual-wavelength photothermal and photodynamic activation (An et al., 2023). In MOFs, photoactive ligands, metal clusters and incorporated agents can similarly support imaging-guided combinations (Sharma et al., 2019; Hu et al., 2022).

Precision depends on light penetration, wavelength, irradiance, duration, thermal diffusion and lesion accessibility. Strong effects in superficial xenografts do not necessarily translate to deep or disseminated disease. Studies should report delivered energy and temperature maps, include light-only and material-only controls, and distinguish photothermal death from photochemical ROS. Near-infrared excitation improves penetration but remains limited in many anatomical sites. Upconversion or X-ray-assisted strategies extend depth at the cost of additional materials and dosimetry.

Ultrasound can penetrate more deeply and activate sonosensitizers or promote cavitation, while magnetic fields can guide iron-containing particles or generate heat. Magnetically aided intracellular delivery of iron-carboxylate MOFs demonstrates the potential to combine carrier composition with external navigation (Sethi et al., 2016). Ultrasound-responsive biomimetic MOFs can also integrate immune modulation (Luo et al., 2022). These systems require clinically relevant field parameters, mapping of off-target energy deposition and an explanation of how heterogeneous tissue properties influence activation. External control is not synonymous with exact control; the energy field and the carrier distribution must overlap at the required scale.

### 6.4 Validating release mechanisms under biological uncertainty

Release assays are often treated as confirmatory measurements, but for a responsive framework they are part of mechanism validation. Dialysis can create an artificial barrier, maintain or violate sink conditions unpredictably and fail to distinguish free drug from drug associated with colloidal fragments. Centrifugation can sediment large particles while leaving degraded nanofragments with the apparent free-drug fraction. Fluorescence may change through quenching, protonation or binding. No single method is universally correct; the separation and detection strategy must be validated for the specific carrier, cargo and transformation products.

A robust study uses orthogonal measurements. Drug can be quantified by chromatography after a separation method shown to recover free and bound fractions. Metal and linker release can be measured in parallel, while diffraction, scattering or spectroscopy reports structural change. Activity assays determine whether a protein or enzyme remains functional after release. Mass balance should be calculated at several time points. If drug release precedes metal or linker loss, diffusion or gate opening is implicated; if all components appear together, framework erosion or collapse is more likely. Mechanistic interpretation should remain conditional when several pathways coexist.

Biological variability should be included early. Serum-protein concentration, phosphate, pH, glutathione, enzymes and cellular uptake vary among species, tissues and disease states. Rather than testing one “normal” and one “disease” condition, experiments should map a response surface. The result can identify a safe operating window and reveal patients or lesions in which the trigger may be insufficient. For externally activated systems, response surfaces should include particle concentration and energy dose. This approach converts a visually appealing trigger into quantitative design information.

Release must finally be linked to pharmacodynamics. A tumor-cell viability curve may reflect drug release, framework toxicity or energy-induced injury. Mechanism-specific biomarkers, target engagement, cargo rescue and inactive analogues can disaggregate these effects. *In vivo*, local free-drug exposure or a validated pharmacodynamic marker is more informative than total lesion-associated drug. The highest evidence tier is achieved when a material attribute predicts release, release predicts target engagement, and target engagement predicts efficacy within the same dose range.

## 7 Therapeutic architectures: from carrier to coordinated pharmacology

### 7.1 Chemotherapy and combination treatment

Frameworks can improve apparent solubility, protect labile drugs, co-load agents and add a second therapeutic function (Zhang et al., 2018; Jia et al., 2019; Fu et al., 2024; Wang et al., 2015; Sharma et al., 2019; Hu et al., 2022; Wang Y. et al., 2024; Han et al., 2023; Zhang et al., 2023; Samanta et al., 2025). These benefits should be separated into carrier effects, framework-derived pharmacology and cargo effects. A combination system is not automatically superior because it contains more functions or produces a stronger endpoint at an unmatched total dose.

The minimum comparator hierarchy should include free drug, unloaded framework, each single component, a physical mixture and, where feasible, an inactive or non-responsive structural analogue. Dose matching must account for metal, framework and cargo exposure; exposure matching may be necessary when the formulation changes pharmacokinetics. Claims of synergy require an interaction analysis rather than the observation that a combination outperforms either component.

Drug resistance provides a rationale for co-localized therapy, but redox-active and catalytic systems may damage normal tissue outside the lesion. Therapeutic index should therefore include organ toxicity, inflammatory markers, delayed effects and catalytic activity at equivalent metal or particle doses. A framework should be retained as a candidate only if its added function produces a measurable advantage over a simpler carrier or clinically relevant formulation.

### 7.2 Phototherapy, chemodynamic therapy and immunotherapy

Photodynamic, photothermal and chemodynamic treatments are compatible with framework chemistry because the scaffold can organize chromophores, metals, substrates and oxygen transport (Tong et al., 2020; Ge et al., 2021; An et al., 2023; Sharma et al., 2019; Hu et al., 2022). The central limitation is not proof of local tumor control under optimized irradiation, but whether light dose, oxygen availability, heat or radical exposure can be controlled in heterogeneous tissue. Phototherapy claims should therefore include dosimetry, tissue geometry, off-target activation and a clinically plausible administration setting.

Immunotherapy introduces a second timescale: acute damage may release antigens and danger signals, whereas durable benefit depends on antigen presentation, T-cell priming, myeloid suppression and memory (Luo et al., 2022). Tumor inhibition alone cannot separate direct cytotoxicity from immune-mediated control. Relevant studies should include immune-cell phenotyping, cytokine profiles, rechallenge or memory assays and toxicity consistent with immune activation.

Parallel advances in non-framework biomimetic nanomedicine reinforce the same spatiotemporal requirement: activatable, tumor-targeting biomimetic nanococktails that coordinate immune-microenvironment reprogramming can extend immunotherapy to low-immunogenic solid tumors (Cui et al., 2024).

### 7.3 Catalytic antioxidant therapy and disease-context switching

Framework catalysis may be directed toward ROS generation in cancer or ROS removal in inflammatory, degenerative, ischemic and fibrotic disease (Duan et al., 2023; Jiang et al., 2023; Xiang et al., 2023). This is a context-dependent activity landscape rather than a fixed class property. Metal valence, pH, substrate concentration, protein binding and antioxidant competition can switch the dominant reaction pathway.

Catalytic claims should therefore report kinetics across physiological and pathological conditions, activity per particle and per metal dose, catalytic persistence and interference by relevant biomolecules. In local wound systems, coupling glucose sensing with antibacterial and antioxidant activity is promising (Yu et al., 2025), but sensing and treatment should not be called autonomous feedback unless sensor output changes dose or activation in real time. Local delivery reduces, but does not remove, the need to quantify metal release, tissue exposure and healing quality.

*In vivo*, this activity landscape is further shaped by endogenous inhibitors that are rarely reproduced in isolated enzyme-mimic assays. First, millimolar intracellular glutathione (GSH) and other thiols act as the dominant antioxidant buffer of tumor and inflammatory microenvironments: they scavenge reactive oxygen species generated by oxidase- and peroxidase-like nanozymes and can reduce redox-active metal centers, so the net therapeutic catalytic output is frequently far lower than buffer-derived catalytic constants predict; nanozymes are therefore increasingly engineered to co-deplete GSH so as to preserve ROS-mediated effects (Ma et al., 2026). Second, adsorption of plasma and cytosolic proteins forms a protein corona that can physically block catalytic sites and inhibit activity, with the effect depending on protein identity and shape (Cong et al., 2024). Third, physiological ionic strength and competing anions (phosphate, bicarbonate, halides) can alter metal coordination and substrate binding. Catalytic claims should therefore be re-measured in the relevant biological matrix rather than in buffer alone and, for tumor applications, interpreted against this glutathione-rich, protein-dense environment before *in vivo* potency is inferred.

## 8 COF architecture as a precision variable

### 8.1 Pore geometry, dimensionality and mechanical behavior

COF drug delivery initially focused on showing that ordered pores could host therapeutics. The next phase is to establish structure-property relationships that predict performance. Three-dimensional polyimide COFs demonstrated drug incorporation in extended porous networks (Fang et al., 2015), while nanoscale two-dimensional systems made dispersibility and cellular access more tractable (Bai et al., 2016). More recent three-dimensional structures with very large pores or defined scu-c topology expand the accessible cargo range and diffusion pathways (Zhao et al., 2023; Das et al., 2022). Yet a larger pore does not invariably improve delivery. It may reduce cargo confinement, increase burst release or weaken the framework. Pore size should be evaluated together with pore connectivity, external surface, solvent accessibility and binding energy.

Particle mechanics may also affect circulation, uptake and tissue penetration. COF composition, layer stacking, thickness and defects can change stiffness, while coatings can add a soft interface. Reviews have begun to connect tunable COF mechanics to nanomedicine (Hu and Lv, 2023), but comparative biological data remain limited. Mechanical characterization should be performed in the hydrated, coated and cargo-loaded state because a dry framework modulus may not describe the particle encountered by cells. If stiffness is proposed as a delivery mechanism, composition and surface chemistry must be held constant while mechanics are varied.

Phenazine-linked and other conjugated COFs show how pore-wall electronics can regulate drug loading through aromatic and charge-transfer interactions (Sasaki et al., 2025). This creates a path toward computationally guided carrier-cargo matching: molecular simulations can rank binding sites and diffusion barriers, then experiments can test loading state, desorption and biological release. The model must include solvent, protonation and competing biomolecules; gas-phase or vacuum binding energies are inadequate. A useful future workflow would combine high-throughput reticular design with a smaller set of hydrated-state and biological assays, narrowing candidates before animal testing.

### 8.2 Surface accessibility and targeted COFs

Targeting a COF requires access to its external surface without sacrificing dispersion or blocking functional pores. Hyaluronic-acid modification has been used to add receptor-associated targeting and sustained release to a two-dimensional COF (Samanta et al., 2025). Other systems use folate, aptamers, peptides or antibodies. The density and spatial distribution of these ligands matter: very high coverage can hinder diffusion, alter charge or increase uptake by off-target scavenger cells. As with MOFs, ligand activity should be measured after incubation in biological fluids, where a corona may mask recognition.

Intrinsic fluorescence or a photoactive backbone can support simultaneous tracking and therapy. Red-fluorescent COF nanospheres were developed for trackable anticancer delivery (Zhang et al., 2023). Tracking is valuable only if signal identity is understood. Fluorescent degradation products may separate from the carrier, and signal persistence can outlast structural integrity. Dual labelling of backbone and cargo, or pairing imaging with chemical quantification, is therefore preferable to a single fluorescence channel. The term theranostic should be reserved for systems in which imaging informs localization, activation, response or dosing, rather than for particles that happen to be fluorescent.

### 8.3 Biodegradable and bioeliminable COFs

The stability that makes COFs attractive can become a clearance problem. Deliberately biodegradable COFs introduce bonds or components that disassemble under physiological or disease-associated conditions (Li et al., 2023). The desired pattern is not simply rapid degradation. A systemic carrier should remain sufficiently stable during formulation and transit, then convert into products whose identity, exposure and elimination are understood. Too-fast degradation causes leakage; too-slow degradation causes persistence; heterogeneous degradation generates a mixture that can be difficult to quantify.

Bioelimination studies should therefore use at least two orthogonal labels or analytical signatures to follow the framework and cargo separately. Total fluorescence, carbon content or a single elemental marker cannot establish the fate of an organic backbone. Radiolabelling, isotope-labelled linkers, mass spectrometry of characteristic fragments and size-resolved chromatography may be combined. Recovered organs should be analyzed over time, including fecal and urinary excretion, with a mass-balance estimate. These experiments are demanding, but without them the claim that a COF is biodegradable remains a chemical observation rather than a pharmacokinetic conclusion.

### 8.4 COF bio-elimination pathways

Renal elimination imposes an additional constraint: a covalently cross-linked network must be broken into fragments small enough to cross the glomerular filtration barrier—typically below ∼5.5 nm hydrodynamic diameter (Soo Choi et al., 2007)—before it can be cleared by the kidney, so biodegradability must be defined not as loss of crystallinity but as generation of sufficiently small, soluble fragments. Two cleavage classes are relevant. Chemically, hydrolytically labile linkages such as boronate esters, imines and hydrazones can be cleaved by protonation-driven hydrolysis, and redox-labile linkages such as disulfides can be reduced by intracellular glutathione, as exemplified by a GSH-triggered degradable porphyrin COF (Li et al., 2023). Enzymatically, cleavage would require that specific backbone bonds be recognized by endogenous hydrolases or oxidoreductases; for most COF backbones this remains an unmet requirement rather than a demonstrated pathway, because no endogenous enzyme has yet been shown to degrade intact COF networks. The route to renal clearance therefore depends on linkage identity, fragment size and solubility, and on the tendency of released aromatic fragments to bind proteins or re-aggregate; bio-elimination studies should report fragment molecular weight and urinary/fecal recovery, with mass balance, before clearance is claimed.

## 9 Expanding disease contexts beyond conventional tumor models

### 9.1 Metabolic and fibrotic disease

Cancer dominates framework nanomedicine because tumors provide recognizable microenvironmental triggers and convenient local-activation models. Metabolic and fibrotic diseases test precision more stringently. They involve diffuse tissue, chronic dosing, multiple cell populations and disease-dependent changes in receptor expression, redox state and extracellular matrix. An effective carrier may need to reach hepatocytes, stellate cells, macrophages or injured epithelium while avoiding long-term metal and particle accumulation. The therapeutic goal is often restoration of homeostasis rather than maximal cell killing.

Metabolic and fibrotic applications illustrate a progression from cell targeting to multi-axis mechanism matching. As one example, a MOF-818@UDPG/ASGR1 system combined a copper-based framework with superoxide-dismutase- and catalase-like activities, an ASGR1 antibody and a metabolic cargo designed to influence S1P/SREBP1c-associated lipogenesis while targeting hepatocytes (Zhang et al., 2026). A related anti-ASGR1 MIL-based nanocomplex paired receptor targeting and ROS handling with thyroid-hormone-driven lipid metabolism for steatotic donor liver (Li et al., 2025), and an integrated nanozyme-exosome platform combined catalytic antioxidant action, vesicle-associated regulatory molecules and ASGR1 targeting in MASLD models (Yu et al., 2026). These examples, drawn in part from the authors’ own program, support the proposition that precision may require aligning cell identity with more than one disease axis. They remain preclinical and should not be interpreted as demonstrating human pharmacokinetics, transplant benefit or chronic safety.

Fibrosis creates another multiscale problem because extracellular matrix impedes penetration while activated fibroblasts, macrophages and injured parenchymal cells exchange profibrotic signals. A MOF-engineered exosome platform reported protection and responsive release of exosomes, catalytic ROS scavenging, antibody-assisted organ targeting and improvement in preclinical multiorgan fibrosis models (Liu et al., 2026). As an illustration of a hybrid design, this represents a conceptual advance over a single-organ, single-cargo system, but “universal” antifibrotic delivery should be avoided as a claim. Lung, liver and kidney have different vascular and cellular barriers, and an antibody module that improves one organ’s exposure may not generalize. Future studies should quantify which cell populations receive intact hybrid particles in each organ and determine whether the same potency mechanism operates across tissues.

### 9.2 Ischaemia-reperfusion injury, neuroinflammation and cardiovascular injury

Ischaemia-reperfusion injury produces rapid ROS generation, mitochondrial damage, ferroptosis, endothelial activation and inflammatory recruitment. The treatment window can be short, making fast distribution or local perfusion more relevant than long circulation. GPX4-containing stem-cell exosomes alleviated ferroptosis and injury in preclinical models of steatotic liver ischaemia-reperfusion (Shi et al., 2025). Although not a framework study, it identifies a functional protein cargo and a disease window that could motivate protective framework or hybrid delivery. Any proposed MOF/COF-exosome integration would need to preserve GPX4 activity, release it in a functional compartment and avoid delaying exposure beyond the reperfusion window.

MOF nanozymes have also been used to regulate neuroinflammation and myocardial oxidative injury (Jiang et al., 2023; Xiang et al., 2023). These applications invert the common tumor-therapy objective: radical scavenging, not radical generation, is desired. The blood-brain barrier, injured vascular permeability and cardiac phagocyte populations create organ-specific delivery constraints. Chiral recognition, cell interactions and catalytic stereoselectivity may add functional precision in neurological disease, but the contribution of chirality should be separated from composition, size and surface charge. Cardiovascular use demands especially careful assessment of thrombosis, complement, haemodynamics and metal-related electrophysiology.

### 9.3 Infection, wounds and local delivery

Local delivery can provide a practical translation route for complex frameworks because it limits systemic exposure and permits high concentrations at an accessible site. Wound dressings, injectable gels, coatings and microneedles can retain MOFs or COFs while allowing drugs, ions or catalytic products to diffuse. The bimetallic MOF nanozyme composite for diabetic wound management combined a wearable glucose-sensing function with microenvironment-dependent catalytic treatment (Yu et al., 2025). This closed-loop concept is compelling: a disease biomarker is measured and the same material family contributes to therapy. A true closed loop, however, would require the sensor output to alter dose or activation in real time; parallel sensing and treatment is an important step but not yet autonomous feedback control.

Antibacterial framework systems frequently generate ROS, release metal ions or deliver antibiotics. Local acidity and bacterial enzymes can serve as triggers, while light or ultrasound can add temporal control. Safety endpoints should include effects on keratinocytes, fibroblasts, endothelial cells, immune cells and the wound microbiome, not only bacterial killing. Healing quality should be assessed through re-epithelialization, vascularization, collagen organization and mechanical recovery. A material that sterilizes a wound but prolongs inflammation or impairs tissue remodelling is not a successful precision therapy.

## 10 Theranostics, quantitative feedback and decision-making

Theranostic frameworks integrate treatment with imaging or sensing. MOFs can carry magnetic, radioactive, photoacoustic, fluorescent or computed-tomography-active components; COFs can encode fluorescence, photoacoustic contrast or photoactivity in the backbone. Pharmacokinetic and biodistribution studies using radiolabelled ZIF-8 demonstrate the value of quantitative whole-body tracking for image-guided delivery (Haripriyaa and Suthindhiran, 2023). Imaging can answer three distinct questions: where the carrier is, whether a trigger has been activated, and whether the disease is responding. Many platforms address only the first. Precision increases when imaging is linked to a decision, such as selecting an activation time, identifying a non-accumulating lesion or adapting subsequent dose. Theranostics therefore contributes most directly to the translational precision dimension of our framework, converting a spatial or mechanistic measurement into a development decision.

Carrier tracking should be chemically interpretable. A label attached to a coating may detach, a metal isotope may persist after framework dissolution, and a fluorescent drug may be quenched while loaded and activated on release. These behaviors can be useful for ratiometric or activatable imaging, but they require calibration. Whenever possible, an intact-particle marker and a release marker should be combined. Imaging-derived concentration should be validated against elemental analysis, chromatography or mass spectrometry in representative tissues.

Sensing can extend precision beyond the particle itself. The authors’ wearable glucose/MOF nanozyme platform links disease monitoring to local wound intervention (Yu et al., 2025). More broadly, framework sensors could measure pH, oxygen, ROS, metabolites or drug exposure and inform when externally triggered systems should be activated. Implantable or wearable devices may be more tractable than fully autonomous injectable feedback systems because they allow signal processing and user control outside the body. For systemic nanomedicine, a realistic near-term goal is image-informed or biomarker-informed dosing rather than a particle that independently diagnoses and treats every lesion. Representative framework strategies and their evidence boundaries are summarized in Table 5.

**TABLE 5:** Representative framework strategies and their evidence boundaries.

Platform or study class	Precision mechanism	Cargo or framework action	Evidence illustrated	Main boundary for interpretation
Iron-carboxylate nanoMOFs	Biodegradable porous carrier	Small-molecule loading and release	Early *in vivo* delivery and toxicology (Horcajada et al., 2010; Baati et al., 2013)	Liver/spleen capture and incomplete long-term component mass balance
PEGylated UiO-66	Surface-controlled stability and uptake	pH-responsive small-molecule release (Abánades Lázaro et al., 2017)	Links PEGylation to dispersion, uptake and release	Surface density, corona masking and chronic Zr/linker fate require definition
Aptamer-functionalized MOF	Biomarker recognition coupled to release	VEGF-associated activation of anticancer drug delivery (Chen et al., 2018)	Target-trigger coupling	Biomarker heterogeneity and *in vivo* trigger specificity
Biomineralized ZIF coating	Cargo protection	Encapsulation of biomacromolecules (Liang et al., 2015)	Protection under stress and release after dissolution	Functional cytosolic delivery and escape are cargo dependent
MOF catalytic cascades	Co-localized enzyme and nanozyme activity	Glucose consumption and ROS generation (Fang et al., 2020)	Spatially organized multistep chemistry	Oxygen/substrate dependence and off-target catalytic exposure
Biomimetic MOF	Membrane-mediated interaction plus physical trigger	Sonodynamic therapy with immune-checkpoint treatment (Luo et al., 2022)	Combines biological interface, organelle stress and immunity	Membrane identity, batch variation and immune mechanism attribution
Nanoscale and 3D COFs	Pore-confined loading	Sustained small-molecule release (Fang et al., 2015; Bai et al., 2016; Zhao et al., 2023; Das et al., 2022)	Demonstrates effect of dimensionality and pore architecture	Dry porosity does not predict hydrated loading or *in vivo* release
Responsive COFs	Linkage- or gate-dependent activation	Acid, redox, hypoxia or light-responsive release (Ge et al., 2021; Anbazhagan et al., 2021; Liu et al., 2020)	Multi-input temporal control	Trigger ranges and incomplete activation in heterogeneous lesions
Photoactive COFs	Framework as carrier and photosensitizer	Photodynamic/photothermal combination (Tong et al., 2020; Wang et al., 2018; An et al., 2023)	Ordered photophysical and loading functions	Light penetration, dosimetry and deep-tissue applicability
Biodegradable COF	Programd backbone disassembly	Combination tumor therapy (Li et al., 2023)	Connects degradability with treatment	Fragment identity, pharmacokinetics and complete elimination remain underdefined
MOF-exosome hybrid	Biological cargo plus framework protection and catalysis	ROS control, metabolic regulation and organ targeting (Liu et al., 2026; Yu et al., 2026)	Multicomponent efficacy in preclinical fibrosis/MASLD models	Requires modular potency assays, intact-hybrid PK and scale-up controls
ASGR1-targeted hepatic MOF/nanocomplex	Receptor-informed hepatocyte exposure	Lipid regulation and antioxidant activity (Zhang et al., 2026; Li et al., 2025)	Matches cell target to metabolic mechanisms	Receptor variability, chronic exposure and human translation are unresolved
MOF nanozyme wound composite	Local adaptive catalysis with sensing	Glucose sensing, antibacterial activity and redox remodelling (Yu et al., 2025)	Accessible, local theranostic use	Parallel sensing-treatment is not yet autonomous dose feedback

## 11 Translation: an evidence chain for safety, fate and manufacturability

### 11.1 Safety cannot be assigned to a framework name

As of the literature search date, no MOF- or COF-based drug product has received marketing approval; however, early clinical translation has begun, led by hafnium-based nanoscale metal-organic framework (nMOF) radiosensitizers. RiMO-301, a hafnium nMOF radiosensitizer, completed a Phase 1 dose-escalation trial combining intratumoral injection with palliative radiation in advanced tumors (NCT03444714, 32 participants) (Koshy et al., 2023), and is now being evaluated in a Phase 1b/2a trial combining intratumoral injection with hypofractionated radiotherapy and a PD-1 inhibitor for unresectable, recurrent or metastatic head-and-neck cancer (NCT05838729, recruiting) (Xie et al., 2026); a related candidate, RiMO-401, entered a Phase 1 trial with radiation in advanced solid tumors but was terminated owing to slow enrolment (NCT06182579). Safety discussions for most framework candidates therefore still rest on preclinical evidence and on general principles drawn from particulate and metal-based medicines. MOF cytotoxicity varies with node, linker, particle size, surface chemistry, dose metric and exposure duration (Ma et al., 2017). In-depth studies of iron-carboxylate nanoMOFs have shown organ sequestration, degradation and excretion patterns that support the feasibility of some compositions (Baati et al., 2013), but these results cannot be generalized to all iron MOFs or to other metals. Recent translational analyzes emphasize a linked evaluation of safety, *in vivo* fate and degradation (Jing et al., 2026; Abbas et al., 2026; Sezgin et al., 2025). A short viability assay establishes only an initial concentration range. A development-oriented package should include haemocompatibility, complement and cytokine responses, immune-cell interactions, immunogenicity (including anti-carrier and anti-PEG antibody responses), genotoxicity and DNA-damage endpoints where relevant, repeat-dose exposure, recovery periods, and organ-specific endpoints determined by distribution.

Compared with clinically approved nanomedicines, framework medicines face a distinct bench-to-bedside gap. Liposomes, lipid nanoparticles and polymeric micelles benefit from decades of scaled, reproducible manufacturing, standardized characterization and established regulatory precedent (Vargason et al., 2021). Framework candidates, in contrast, must additionally define node/linker stoichiometry, defect density, hydrated stability, degradation-product identity and multi-component dose metrics—attributes that are only now being standardized and that a handful of early clinical candidates (locally injected radiosensitizers) are beginning to test (Koshy et al., 2023; Xie et al., 2026). This comparison underscores that the principal translation bottleneck is reproducible, characterizable manufacture and component-resolved safety, rather than demonstration of *in vivo* activity alone.

The correct dose denominator is not always particle mass. Metal dose, linker dose, cargo dose, particle number, surface area and catalytic activity can each influence toxicity. Reports should provide enough information to convert among these quantities. Framework-only controls are essential, and degradation products should be tested at exposure-relevant concentrations. If a polymer, antibody, membrane or vesicle is added, its impurities, immunogenicity and batch variability become part of the safety assessment. For photo- or sono-active systems, energy dose and material dose form a joint exposure.

COF biocompatibility presents a different but equally demanding problem. Organic composition avoids some metal-ion concerns but introduces monomer, catalyst, solvent and persistent-backbone questions. Reviews of COF nanomedicine repeatedly identify inconsistent morphology, hydrophobic aggregation, limited degradation data and sparse long-term *in vivo* evidence (Zhong et al., 2025; Hu W. et al., 2026; Zhao et al., 2026; Kolodziejczyk and Plonska-Brzezinska, 2026; Kaur et al., 2024). A chemically stable COF may be well tolerated in a short experiment and still persist. Conversely, an unstable COF may release reactive aromatic or aldehyde-containing fragments. Safety must be measured on the final, cargo-loaded, coated product and its transformation products.

### 11.2 Pharmacokinetics and component-resolved fate

Framework pharmacokinetics should distinguish intact carrier from released components. Total metal measured by inductively coupled plasma mass spectrometry does not indicate whether a MOF remains intact; fluorescence can detach; and linker metabolites may follow a different route from the node. At minimum, two product components should be tracked over time. Blood sampling should quantify early distribution and terminal phases, while tissue analysis should include liver, spleen, kidney, lung and lesion as appropriate. Excretion and recovery improve interpretation of whether declining tissue signal reflects elimination, redistribution or loss of the label.

Particle dissolution can make conventional noncompartmental pharmacokinetics difficult because the entity changes during observation. A useful model may include intact particle, circulating fragment, soluble metal or linker and released drug compartments. The relevant exposure-response relationship might be lesion-associated intact particle for phototherapy, intracellular released drug for chemotherapy, or catalytic activity for a nanozyme. The pharmacokinetic analyte should follow the mechanism of action. Radiolabelled ZIF-8 work illustrates how quantitative tracking can improve interpretation (Haripriyaa and Suthindhiran, 2023), but no single label can solve the speciation problem.

### 11.3 Chemistry, manufacturing and controls

Scale-up can alter nucleation, mixing, heat transfer, particle-size distribution, defect density and residual solvent. A robust process begins with a quality target product profile: route, dose form, size range, loading, release, sterility, storage and intended mechanism. Critical material attributes may include node-to-linker ratio, crystallinity, defect markers, hydrated size, aggregation, surface ligand density, free cargo, cargo activity, residual reagent, endotoxin and water content. Critical process parameters may include addition order, mixing energy, temperature, pH, modulator concentration, purification and drying.

Batch release should rely on orthogonal assays. Powder X-ray diffraction alone does not establish nanosuspension identity; thermogravimetry alone does not distinguish free and bound cargo; and total loading does not predict release. A practical panel may combine diffraction, spectroscopy, microscopy, particle sizing, composition, residual-solvent analysis, free-drug quantification, a release assay and a mechanism-linked potency assay. For catalytic products, activity under standardized substrate and pH conditions is a potency attribute. For hybrid exosome or membrane products, vesicle identity, cargo markers, membrane orientation and biological activity must be added.

Sterilization and storage are often deferred until late development. Filtration may be impossible for larger particles; heat, irradiation or chemical sterilants can change frameworks, linkers, drugs and biological coatings. Aseptic manufacture may therefore be required. Lyophilization can improve storage but induce aggregation or pore changes unless a cryoprotectant is selected. Stability studies should monitor not only appearance and size but also cargo leakage, framework transformation, coating integrity and potency. Complexity is acceptable only when these attributes remain within specifications.

### 11.4 Synthetic scale-up challenges for framework medicines

Scaling framework medicines from laboratory synthesis to reproducible manufacture faces challenges that are distinct from those of conventional polymers or lipids. Many MOFs and COFs are prepared in dilute solvothermal batches using high-boiling organic solvents that must be removed to pharmaceutical limits; particle size, defect density and residual solvent can drift between batches when mixing, heating and cooling rates are not tightly controlled. Continuous-flow synthesis offers a route to tighter residence-time and temperature control and to higher throughput (Dunne et al., 2016). Green or aqueous solvent systems reduce the downstream purification burden and are increasingly considered together with scale-up strategy, waste, energy use and techno-economic feasibility (Singh et al., 2026). Finally, activation and drying are not trivial steps: the activated porosity that underpins loading capacity depends sensitively on solvent exchange and on thermal or supercritical treatment, and incomplete activation leaves residual solvent in the pores that reduces usable capacity. The relevant benchmark for drug delivery is therefore a reproducible, sterile, endotoxin-controlled nanosuspension with defined size, loading, free fraction and stability, rather than gram-scale powder synthesis alone.

### 11.5 Regulatory strategy and development decisions

Framework medicines will usually be assessed as complete drug products rather than as abstract material classes. The development path depends on which component produces the principal pharmacological action. A MOF carrying an approved small molecule may still differ substantially from the free drug because distribution and degradation change exposure. A catalytic MOF, photoactive COF or framework-drug conjugate may function as a new active substance. A membrane- or exosome-coated framework adds biological starting materials and process controls. Combination with light, ultrasound, a wearable sensor or a perfusion device can create a drug-device or biologic-drug combination problem. These distinctions should be discussed with regulators before the pivotal nonclinical package is fixed.

A practical development program uses stage gates. At the candidate gate, composition, impurities, hydrated-state behavior, potency and preliminary safety must be reproducible across independent batches. At the proof-of-mechanism gate, delivery and pharmacodynamic endpoints must separate framework, cargo and combination effects. At the development-candidate gate, a scalable process, stability-indicating assays, route-appropriate pharmacokinetics and a justified dose metric are required. At the investigational gate, toxicology material should be representative of the proposed clinical process, and the analytical methods should be capable of detecting changes that matter to function.

The impurity strategy deserves specific attention. Residual modulators, metals, catalysts, solvents, unreacted monomers, endotoxin and free drug may each contribute to biological effects. Acceptable limits should be linked to toxicology and process capability, not chosen only because an analytical signal is small. For COFs, residual aldehydes, amines and catalyst species can be relevant; for MOFs, soluble metal and unbound linker require control. Biological coatings introduce host-cell proteins, nucleic acids, lipids and potential adventitious agents. Purification must remove impurities without stripping the functional interface or destabilizing the framework.

Reference standards are also challenging because some products transform during analysis. A well-characterized framework batch, a cargo standard, degradation-product standards and an activity reference may all be needed. Assays should include system-suitability controls that distinguish analytical drift from product change. For a nanozyme, catalytic activity should be traceable to standardized conditions; for a photoactive COF, optical absorption, photothermal conversion or reactive-oxygen yield should be related to an exposure protocol; for a targeted product, ligand accessibility and receptor binding should be measured on the final formulation.

Clinical translation should begin with an indication and administration setting that exploit framework strengths while containing risk. Accessible lesions, local delivery, isolated-organ perfusion or short-course therapy may offer clearer exposure control than chronic systemic dosing. The authors’ donor-liver nanocomplex work suggests isolated perfusion as one such setting (Li et al., 2025), while wound platforms provide another (Yu et al., 2025). This does not guarantee an easier regulatory path, but it can make distribution, retrieval, activation and response more measurable. Indication selection is therefore part of precision design. The translational evidence ladder is shown in Figure 3.

**FIGURE 3:**
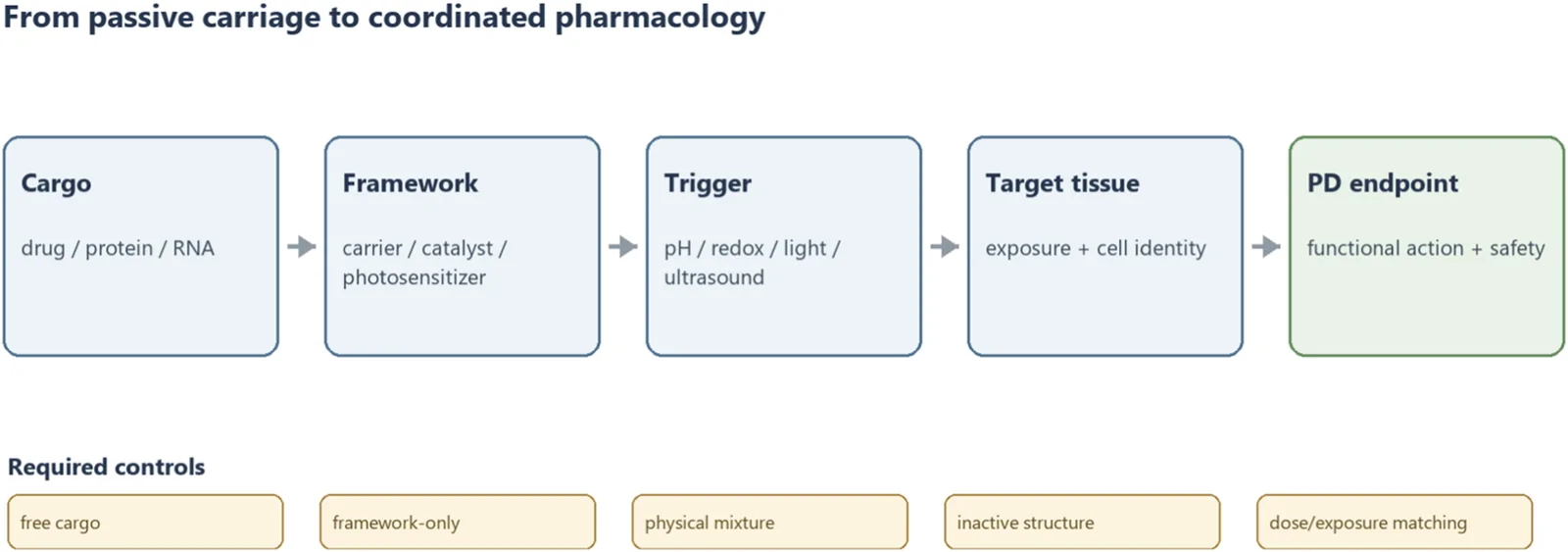
Translational evidence ladder for MOF and COF medicines. A five-level ladder progresses from material proof (composition, structure and loading), through biological feasibility (stability, uptake and release), mechanistic pharmacology (target engagement and functional cargo), quantitative *in vivo* development (PK, biodistribution, degradation and dose-response), to translation readiness (repeat-dose safety, scalable CMC, storage, sterility and clinically relevant administration). Each level lists disqualifying gaps. A side branch shows that adding a targeting ligand, imaging agent, membrane or exosome adds a parallel set of critical quality attributes rather than automatically moving the product upward.

## 12 Design rules and research agenda

First, begin with a delivery bottleneck, not a framework. The initial specification should identify route, target cell, subcellular destination, therapeutic window and principal barrier. A framework should be selected because its pore chemistry, degradation, catalytic function or photophysics solves that barrier. If a conventional liposome, polymer particle or simple conjugate provides the required function with lower analytical burden, it is the stronger translational choice.

Second, define the active entity and its dose. For a passive carrier, the active entity may be released drug. For a nanozyme it may include catalytic sites; for a photoactive COF it may be the intact framework under illumination; for a degradable MOF it may change over time. Dose and potency assays should reflect that definition. Multicomponent hybrids require modular and integrated potency tests so that loss of one function is detectable.

Third, couple cargo affinity to the intended release pathway. Loading should be optimized against leakage and target-site release, not maximized in isolation. Computational predictions, hydrated-state measurements and biological release experiments should converge on the same mechanism. Pore-blocking coatings and ligand conjugation must be assessed after cargo loading, because the final order of assembly can change access.

Fourth, measure precision at the claimed spatial scale, as defined in Section 2: organ targeting requires quantitative organ exposure, cell targeting requires cell-resolved delivery, and cytosolic delivery requires a functional intracellular readout. Histological colocalization is supportive but not sufficient by itself. Receptor-blocking, target-negative and disease-stage controls should accompany active targeting.

Fifth, treat the biological interface as a dynamic component. Protein corona, membrane coating, polymer shedding and vesicle integrity can change during circulation. Characterization should therefore be time-resolved and performed in relevant fluids. Biomimetic coatings should be defined by source, purification, orientation and retained function, not only by the presence of membrane proteins.

Sixth, separate trigger response from framework degradation. Studies should quantify intact structure, metal or backbone fragments, and cargo simultaneously. Trigger conditions must be physiologically plausible and compared with normal-tissue conditions. Externally activated systems should report dosimetry and tissue geometry.

Seventh, build safety and manufacturability into candidate selection. Toxicological screening should include every component and route-specific exposure. Early scale-down models of mixing, purification, sterilization and storage can reveal whether a sophisticated architecture is controllable. Candidate selection should penalize poorly defined defects, toxic solvents, irreproducible coatings and dose metrics that cannot be standardized.

The research agenda follows from these rules. Shared reference materials and reporting standards are needed for representative MOF and COF families. Interlaboratory studies should compare particle sizing, loading, release and catalytic assays on the same batches. Dual-labelled pharmacokinetic experiments should become routine for degradable frameworks. Human-relevant models, including organoids, perfused tissues and immune-competent systems, can test barrier and toxicity mechanisms before extensive animal use. Data-driven design should integrate synthesis parameters, structural descriptors, hydrated-state behavior and biological outcomes, while preserving negative and failed formulations to avoid biased models. Most importantly, studies should define a pre-specified success criterion that connects a material attribute to a pharmacological decision.

### 12.1 Minimum evidence packages for different precision claims

A shared vocabulary would reduce overclaiming; Table 1 summarizes the claim-to-evidence mapping and essential controls. A responsive carrier claim should require reproducible trigger-dependent release under biologically relevant conditions, with normal-condition controls and simultaneous assessment of carrier transformation. A targeted carrier claim should require quantitative receptor-dependent delivery at the stated spatial level, plus pharmacokinetic controls showing that the effect is not explained only by altered circulation. A functional macromolecule delivery claim should require sequence-, activity- or rescue-based evidence after intracellular release. A theranostic claim should require that the diagnostic signal informs localization, activation, response or dose. A biodegradable claim should require identification and time-resolved disappearance of the intact carrier, not only loss of crystallinity in buffer.

For an early cell study, the minimum package includes final-formulation composition, size in complete medium, cargo loading and free fraction, time-resolved uptake, trigger or release validation, framework-only and free-cargo controls, and a functional endpoint. For a first animal study, the package should add blood compatibility, tolerability, quantitative organ and lesion distribution, target-cell evidence where claimed, dose-response, pharmacodynamic biomarkers and short-term component fate. These are not substitutes for formal safety studies; they prevent efficacy experiments from being interpreted without basic exposure information.

For a translational candidate, the package expands to multiple batches, stability, sterility strategy, endotoxin, scalable purification, validated potency, component-resolved pharmacokinetics, degradation and excretion, repeat-dose toxicology when relevant, immunogenicity for biological coatings, and administration-device compatibility. The same formulation should be used across pivotal efficacy, pharmacokinetic and safety experiments or bridging should be justified. Changes in particle size, coating or loading can change biological behavior enough that a nominally identical framework name does not ensure comparability.

Negative results should be incorporated into design rules. A formulation that aggregates in serum, loses its ligand, binds drug too strongly or shows liver persistence is informative if its attributes are reported completely. Publishing only the best-performing formulation creates an artificial relationship between structure and efficacy and undermines machine-learning approaches. Pre-registration is not required for every material-screening study, but prospective success criteria and transparent reporting of screened candidates would improve the field’s cumulative value.

Interlaboratory reproducibility should be treated as a biological performance test, not only as a chemistry exercise. The same nominal framework prepared with different water content, mixing history, modulators or purification can differ in defect density, residual reagents, size and dissolution. A useful round-robin study would distribute common starting materials and a shared protocol, then compare both physicochemical attributes and a small set of biological endpoints. A second phase would distribute one centrally manufactured batch to separate analytical laboratories, distinguishing manufacturing variation from measurement variation. Reference dispersions with defined aggregation and degradation states could help qualify particle-sizing and release methods. These studies would identify which measurements are sufficiently robust to become specifications and which remain exploratory. They would also provide realistic uncertainty intervals for structure-property models. Precision delivery cannot be more reproducible than the assays used to define it; therefore, method transfer, operator sensitivity and data-processing choices should be documented alongside particle performance.

Finally, precision should be tested against a simpler benchmark. The appropriate comparison may be free drug, a clinically used formulation, a conventional nanoparticle or the same targeting ligand on a non-framework carrier. A framework that achieves similar efficacy with a lower dose, wider safety margin, uniquely protected cargo or controllable activation has a defensible value proposition. A framework that only increases loading relative to an unoptimized comparator does not. This comparative discipline will narrow the enormous design space to candidates with plausible clinical utility and regulatory review readiness.

### 12.2 Limitations of this review

This is a narrative, mechanism-organized review rather than a systematic review or meta-analysis; study selection and evidence tiering therefore depend on author judgment, and the inclusion and exclusion decisions described in Section 1.1 cannot eliminate selection bias. In particular, six of the illustrative worked examples are drawn from the authors’ own preclinical program (Liu et al., 2026; Zhang et al., 2026; Li et al., 2025; Yu et al., 2026; Shi et al., 2025; Yu et al., 2025). These studies are presented as examples of the framework’s design logic rather than as independently replicated evidence, and we acknowledge that their prominence may bias which platforms are foregrounded as exemplars. The literature is also dominated by cell and small-animal studies, and the quantitative benchmarks we advocate (tumor delivery efficiency, component-resolved pharmacokinetics and degradation mass balance) remain sparse for many framework families; where we cite quantitative anchors, they derive from the referenced primary studies rather than from a formal meta-analysis.

## 13 Conclusion

MOFs and COFs provide an unusually programmable bridge between molecular design and mesoscale drug delivery. Their pores can concentrate and protect cargo; nodes and linkers can create responsive, catalytic, magnetic or photoactive functions; and external surfaces can be equipped with polymers, ligands, membranes or extracellular vesicles. The resulting design space supports therapies that are difficult to achieve with a passive carrier, including reaction cascades, multi-input release, framework-derived phototherapy, antioxidant nanozymes and integrated biological-synthetic hybrids.

The same programmability increases the burden of proof. Precision cannot be inferred from porosity, a targeting ligand or a triggered release curve. It must be demonstrated as a linked sequence from reproducible material identity to route-appropriate stability, quantitative exposure, target-cell and subcellular delivery, functional release, therapeutic mechanism, safety and clearance. Current evidence is rich in material feasibility and preclinical efficacy but thinner in component-resolved pharmacokinetics, long-term toxicology, clinically relevant administration and scalable manufacturing.

The most productive future is therefore not an indiscriminate expansion of framework complexity. It is precision by design: choose the simplest reticular architecture that addresses a defined biological bottleneck, encode only functions that can be measured, and advance candidates through an evidence ladder that makes failure informative. Under this discipline, MOFs and COFs can evolve from impressive porous materials into accountable drug products whose chemical programmability is matched by pharmacological and manufacturing precision.
